# Acquire continuous and precise score for fundus image quality assessment: FTHNet and FQS dataset

**DOI:** 10.1038/s41598-025-24423-8

**Published:** 2025-11-18

**Authors:** Zheng Gong, Zhuo Deng, Run Gan, Zhiyuan Niu, Lu Chen, Canfeng Huang, Jia Liang, Weihao Gao, Fang Li, Shaochong Zhang, Lan Ma

**Affiliations:** 1 Tsinghua University, Tsinghua Shenzhen International Graduate School, Shenzhen, China; 2 Shenzhen Eye Hospital, Shenzhen, China

**Keywords:** Image processing, Eye diseases

## Abstract

The retinal fundus images are extensively utilized in diagnosis, and their quality may affect diagnostic results. However, due to limitations in the datasets and algorithms, current fundus image quality assessment (FIQA) methods often lack the granularity required to meet clinical demands. To address these limitations, we introduce a new benchmark FIQA dataset, Fundus Quality Score, which contains 2,246 images annotated with continuous mean opinion scores ranging from 0 to 100 and three-level quality categories. Meanwhile, we also design a novel FIQA Transformer-based Hypernetwork (FTHNet). The FTHNet can treat FIQA as a regression task to predict the continuous MOS, diverging from common classification-based approaches. Results on our dataset show that FTHNet predicts quality scores, achieving a Pearson Linear Correlation Coefficient of 0.9423 and a Spearman Rank Correlation Coefficient of 0.9488, significantly outperforming compared methods while utilizing fewer parameters and lower computational complexity. Furthermore, model deployment experiments demonstrate its potential for use in automated medical image quality control workflows. We have released the code and dataset to facilitate future research in this field.

## Introduction

The retinal fundus image is one of the most commonly used ophthalmology graphics. Many ophthalmologists use fundus images to assist clinical diagnosis of diabetic retinopathy (DR)^1–3^, age-related macular degeneration (AMD)^4^, polypoidal choroidal vasculopathy (PCV)^5^, and other retinal diseases^6,7^. The precise diagnosis of eye diseases relies on high-quality(HQ) fundus images. However, fundus images captured with different equipment by ophthalmologists with various levels of experience have large variations in quality. A screening study of 5,575 patients found that about $12\%$ of fundus images are of inadequate quality to be readable by ophthalmologists^8^. Moreover, another study based on the UK BioBank also shows that more than 25$\%$ of fundus images need to be of higher quality to allow accurate diagnosis. Consequently, low-quality(LQ) fundus images cover a significant percentage of clinical fundus images. According to the experience of ophthalmologists, the common degradation types of LQ fundus images include out-of-focus blur, motion blur, artifact, over-exposure, and over-darkness. The degradation of fundus images may prevent a reliable clinical diagnosis by ophthalmologists or computer-aided systems. Thus, fundus image quality assessment (FIQA) is proposed to help ophthalmologists control the quality of fundus images. The FIQA tasks can be bonded with the collection process of fundus images, which can boost its speed and avoid useless repeats. Moreover, the quality control process in the medical record system can also benefit from FIQA methods. Thus, the research in FIQA is important.

Traditional FIQA methods^9–11^ primarily rely on hand-crafted models. However, these approaches often achieve unsatisfactory performance and limited generalizability. In recent years, Convolutional Neural Networks (CNNs) have been widely adopted in image quality assessment (IQA)^12–15^. Inspired by the success of IQA, CNNs have likewise been applied to FIQA^16,17^. While achieving impressive results, CNN-based methods struggle to capture long-range dependencies effectively. More recently, the Transformer architecture^18^ was introduced to computer vision and has surpassed CNN-based methods in numerous tasks. The Multi-head Self-Attention (MSA) mechanism within the Transformer excels at modeling non-local similarity and long-term dependencies. This inherent strength of the Transformer has the potential to overcome the limitations observed in CNN-based models. Consequently, Transformer-based methods have been extensively applied in fundus-related tasks, such as disease screening^19–21^, focus segmentation^22,23^, and image restoration^24,25^. The compelling performance of the Transformer architecture also demonstrates significant potential for the FIQA task. Therefore, we selected the Transformer as one of the foundational feature extraction architectures for our model.

Meanwhile, most present FIQA methods are data-driven, which means the performance of these methods relies on the quality of the data. Unfortunately, a professional and available clinical benchmark has yet to be explored. **Firstly**, some datasets^16,26,27^ treat the FIQA task as a classification task rather than a regression task. The fundus images are roughly divided into several categories, such as “Good”, “Usable”, and “Bad” quality. The roughly labeled images can lead to images of prominently different quality classified in the same category. **Secondly**, when a quality scale is used, some datasets do not consider that the image quality of different fundus regions has different effects on clinical diagnosis. For example, the influence of optic disc regions on the fundus image quality should be more significant than that of edge regions. **Thirdly**, many works train and test on their private datasets, which are labeled using their own standard and are not publicly available. Therefore, it is not convenient to benchmark the performance of the FIQA methods.

**Fig. 1:**
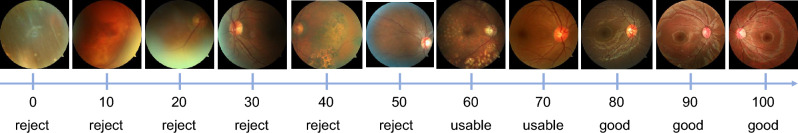
Examples of the Fundus Quality Score (FQS) dataset. In the FQS dataset, each fundus image has two labels: a three-level classification label (good, reject, and usable) and a continuous MOS varying from 0 to 100. Our FQS dataset covers the most common degradation types in clinical diagnoses, such as out-of-focus blur, haze, uneven illumination, and over-darkness.

In this paper, we set out to address the limitations of algorithms and datasets in FIQA. To begin with, we establish a new clinically acquired dataset, Fundus Quality Scores (FQS), including 2246 fundus images with continuous mean opinion scores (MOSs) ranging from 0 to 100 and three-level labels (“Good”, “Reject”, and “Usable”). Based on this dataset, we propose a novel method for FIQA, namely the FIQA Transformer-based HyperNetwork (FTHNet). The proposed FTHNet consists of four parts: the Transformer Backbone, the Distortion Perception Network, the Parameter Hypernetwork, and the Target Network. Specifically, the Transformer Backbone is built up by Basic Transformer Blocks (BTBs). The self-attention mechanism in BTBs can capture the non-local self-similarity and long-term dependencies, which are the main limitations of existing CNN-based methods. The proposed Distortion Perception Network can collect distortion information in different resolutions. We introduce the Parameter Hypernetworks, which can dynamically generate weights and biases according to fundus image contents. Furthermore, the Target Network receives the weights and biases and predicts fundus image quality scores. This proposed method is supposed to give prediction scores consistent with ophthalmologists’ experience and perception.

Our contributions can be summarized as follows:

- We establish a new clinical dataset, FQS, to evaluate FIQA algorithms. This is the first professional and available FIQA dataset with both continuous MOSs and three-level classification labels.
- We propose a novel FIQA model with the Transformer-based hypernetwork, FTHNet. It is the first attempt to introduce the Transformer, aligning with the hypernetwork for FIQA tasks.
- Experimental results demonstrate that our FTHNet significantly outperforms current algorithms in the FIQA tasks with fewer parameters and less computation complexity, and thus has excellent potential in real-time diagnosis assistance.

## Material and methods

### Fundus quality score dataset

This subsection details the Fundus Quality Score (FQS) clinical dataset, which contains 2246 fundus images. The original images possess spatial dimensions approximating $1942\times 1942$ pixels and were standardized to $1024\times 1024$ pixels for consistent processing. Each image is accompanied by two labels: a categorical quality rating (’Good’, ’Usable’, ’Reject’), and a Mean Opinion Score (MOS). The MOSs represent perceiving image quality on a continuous scale from 0 (minimum) to 100 (maximum). The data is available at https://figshare.com/articles/dataset/FIQS_Dataset_Fundus_Image_Quality_Scores_/28129847?file=51531041, which can be downloaded and utilized in reproduction and any other FIQA research. The samples of this dataset are shown in Fig. 1.

#### Data collecting

We include all fundus images collected from the partner hospital between December 2021 and Jun 2022. Over 10,000 eye instances were collected during this period. To compile a dataset reflecting the natural distribution of image quality and degradation types, all degradation types were maintained for fundus images encountered in clinical practice. Subsequently, duplicate images and images of similar content and quality were excluded from the dataset. The data duplication and potential leakage in our dataset have been carefully managed during the selection process. The same images will not appear in different sub-datasets. Therefore, **2246** images were left after the collection, selection, and exclusion. These raw images have spatial sizes of $2656 \times 1992$. Following image acquisition, the preprocessing was applied to these images. Initially, black peripheral borders were removed from each fundus image. This cropping procedure resulted in images with dimensions of $1942\times 1942$ pixels. To ensure maximum fidelity and prevent data loss, these processed images were subsequently saved in the lossless PNG format. Finally, to standardize input size and improve computational efficiency in subsequent analyses, all images were then resized to $1024\times 1024$.

The fundus images are captured by ophthalmologists using a ZEISS VISUCAM200 fundus camera or a Canon fundus camera, which are the mainstream products of fundus cameras. Sensitive information, such as names and diagnosis results, is deleted from the beginning of data collection. The EXIF metadata contains other data points that could cause risks to confidentiality, which are thus deleted. It can include: GPS coordinates, timestamps, and device identifiers. These data elements could inadvertently lead to the re-identification of patients, particularly if correlated with other available information. Therefore, to minimize any potential risk of identity leakage, we removed all EXIF metadata from every image included in the FQS dataset. This action aims to ensure patient anonymity and wider research use without compromising privacy.

**Fig. 2:**
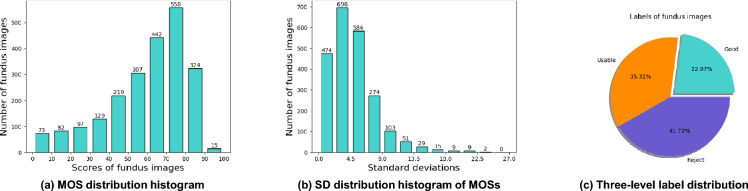
The statistical information of our FQS. (**a**) The MOS distribution histogram. Most of the MOSs are distributed between 60 and 80, which is consistent with actual clinical experience, and images of either extremely high or low quality are rare. (**b**) The standard deviation distribution histogram of MOSs. Half of the images have standard deviations under 4.34. Low SDs indicate that, though the opinion scores are given independently, the scoring criteria are consistent. (c) The three-level label distribution. The numbers of ‘Good’, ‘Usable’, and ‘Reject’ are 516, 793, and 937. There are more ‘Reject’ images to cover more degradation types.

The collection process of our FQS dataset is approved and supervised by Shenzhen Eye Hospital. This study was conducted in accordance with the ethical standards of the institutional and national research committee and with the 1964 Helsinki declaration and its later amendments under supervision. The study protocols and experimental procedures for the collected data were approved by the Institutional Ethics Committee of Shenzhen Eye Hospital (ETHICAL NUMBER: 2022KYPJ062). The use of historical clinical data met the following criteria: (1) All personally identifiable information was removed to ensure patient anonymity and privacy. (2) The research posed minimal or no risk to the subjects both during and after the study. (3) Patients provided consent for their data to be used in future research during the initial data acquisition. Therefore, a formal waiver of written consent was granted by the Institutional Ethics Committee for this specific research project (Fig. 2).

**Fig. 3:**
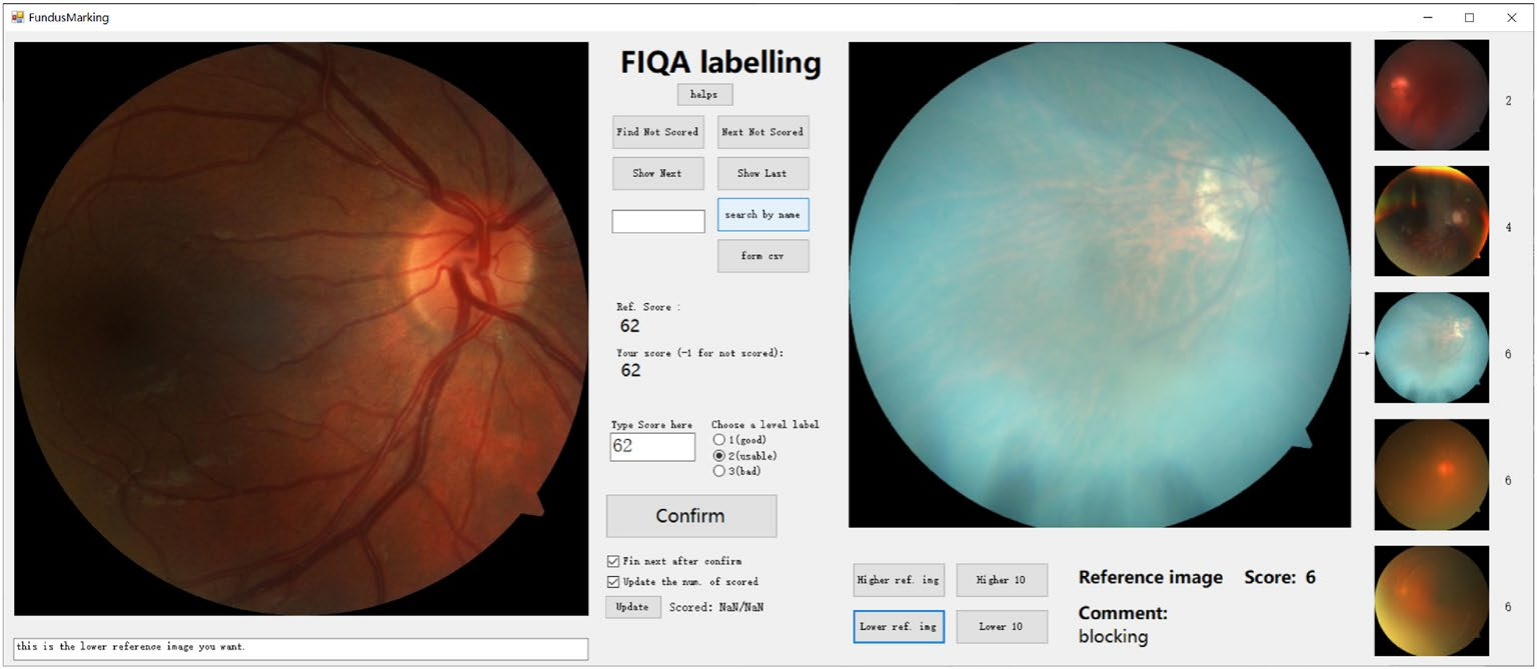
The GUI of our FundusMarking software. This software was developed during the labeling of the FQS dataset for the better convenience of ophthalmologists. The graphical user interface (GUI) of the FundusMarking software is designed to be straightforward. We have released the code for this tool. Thus, it can be easily modified to build any new IQA datasets.

#### Labelling

**First**, a reference set of 330 fundus images was established. Experienced ophthalmologists assigned each image to one of three broad quality categories: “Good,” “Usable,” or “Reject.” This initial classification was based on their clinical expertise and aligns with quality control procedures existing at the cooperating hospital. Correspondingly, initial score ranges were assigned: ’Good’ (80–100), ’Usable’ (60–79), and ’Reject’ (0–59), reflecting a common 100-point quality assessment paradigm.

**Second**, within each broad category, images were further leveled by the ophthalmologists into five classes based on the severity of image degradation (e.g., extent of out-of-focus blur, haze, uneven illumination). This step refined the initial broad score ranges into more detailed sub-ranges. For instance, images initially in the “Good” (80–100) category could be further refined into five classes, 100-95-90-85−80, based on this detailed assessment.

**Third**, the scores derived from the second stage were then subject to a final fine-tuning adjustment. Experts considered the following four aspects to adjust the score, ranging in ±2 points:

1. Comparative degree of fundus image degradation relative to other images within the same refined sub-category.
2. The extent and clarity of discernible structural information for diagnosis (like blood vessel morphology and density).
3. The presence and assessability of pathological features relevant to clinical diagnosis.
4. The visibility and clarity of critical anatomical landmarks (like macula, optic disc).

This adjustment mechanism can differentiate images with intricate quality differences, resulting in the final score values ranging from 0 to 100 with a minimum resolution of 1 point.

The final MOS for each image was determined by weighted averaging the scores from six experts. This approach is common in the development of image quality assessment datasets, such as KonIQ-10k^28^, LIVE^29^, CSIQ^30^, and TID2013^31^, which also utilize MOS to capture perceived quality. Then, three ophthalmologists and three experienced ophthalmologists gave their opinion scores for 2246 images. To make the labeling process more convenient, we built the software FundusMarking. This software has a straightforward GUI and can be easily modified to build any new IQA datasets. The sample of its GUI is shown in Fig. 3. The codes have been released at https://github.com/HudenJear/FIQA-GUI-Labeling. Note that scores will be discussed and adjusted if there is a significant difference between the opinion scores of the same image.

**Finally**, we obtain the MOS by the weighted average of six independent scores:

1 $$\begin{aligned} \textbf{MOS} = \lambda _{1}\sum _{i=1}^{3}\textbf{O}_{i} + \lambda _{2}\sum _{i=1}^{3}\textbf{Oj}_{i}, \end{aligned}$$

where $\textbf{O}_i$ and $\textbf{Oj}_i$ denote the opinion scores of ophthalmologists and experienced ophthalmologists. $\lambda _1$ and $\lambda _2$ represent the weights of ophthalmologists and experienced ophthalmologists. To reflect the greater experience of the senior ophthalmologists, we assigned each experienced ophthalmologist double the weight of an ophthalmologist. As a result, the weight of ophthalmologists and experienced ophthalmologists can be 1/9 and 2/9.

#### Dataset splitting

The train, validation, and test subsets are split proportionately 80%, 5%, and 15% randomly. We have applied a strict inclusion and exclusion standard, so that all images will not be replicated or similar. Therefore, there will be only one image included in the datasets for all images captured during a short period of time for one patient. There is no chance that the same individual’s images for the same eyes may fall into different datasets. However, the two images from the same individuals but from different eyes (left and right) might be included in different datasets (train, validation, or test). As they have completely different features in image quality, it will not cause data leaking across datasets. To definitively prevent such data leakage and adhere to best practices in medical image analysis, we ensure that our random dataset splits are performed at the image level. This means that images will not be replicated in the datasets.

#### Statistic information

The FQS dataset comprises 2246 fundus images in total. Specifically, 92% of selected images are from the usual clinical diagnosis across all ages and genders, and the other 8% are from teens’ myopia screening. The mean and standard deviation of age are 45.24 and 25.17. Regarding eye laterality, the dataset contains 1141 images of the right eye and 1105 images of the left eye. The images were obtained from a cohort of 1170 male and 1076 female patients. The geographic origin of this patient cohort is predominantly East Asian (99.55%), with smaller representations (0.45%) from Central and Southeast Asia. We also summarize more statistical information in the following figures and description.

**MOS Distribution Histogram** Figure 2(a) depicts the distribution of MOS. It can be observed that most of the MOSs are distributed between 60 and 80. The number of images with MOS distributed between 70 and 80 is the largest, and the number of images from 90 to 100 is the lowest, with only 15. The MOS distribution in our FQS is natural and consistent with actual clinical experience. Affected by the equipment and patient coordination, it is difficult to obtain very high-quality fundus images (MOS: 80 or above) in actual clinical collecting. Meanwhile, retinal fundus images of extremely low quality are also rare. The quality of most clinical fundus images is in the upper middle of the score scale.

**Three-level Label Distribution** The distribution of the three-level classification label is shown in Fig. 2(c). Since the HQ images are similar, but the LQ images are different in numerous aspects, there are more “Reject” images in the FQS dataset to cover more degradation types. The numbers of fundus images labeled “Good”, “Usable”, and “Reject” are 516, 793, and 937, respectively. The distribution of the three-level classification label is consistent with the MOSs, which is common in actual clinical fundus image collecting.

**Standard Deviation Distribution Histogram** Since the MOS of each fundus image consists of multiple independent opinion scores from several ophthalmologists, it is necessary to evaluate the uniformity of these six scores. The standard deviation (SD) can present the difference between opinion givers for each. Therefore, we calculate the SD of each image and make the following SD distribution histogram (Fig. 2(b)). About a quarter of the SDs are less than 2.52, half of the SDs are under 4.34, and three-quarters are under 6.37. Thus, the distribution of SD indicates the consistency of opinion scores and proves that prior knowledge of the labeling process is consistent between opinion givers.

**Fig. 4:**
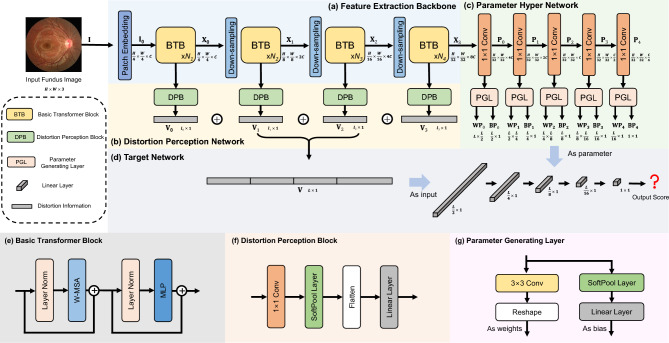
Architecture of our FTHNet. (**a**) The Transformer Backbone includes a patch embedding layer and four feature extraction stages. (**b**) The Distortion Perception Network is designed to extract distorted information. (**c**) The Parameter Hypernetwork comprises five parameter-generating layers. (**d**) The Target Network contains five linear layers to predict the fundus image quality scores. (**e**) The structure of the Basic Transformer Block. (**f**) The distortion perception block extracts the distortion information from the feature maps in different resolutions. (**g**) Each parameter-generating layer includes two branches to generate weight and bias parameters.

### Method

#### Overall architecture

The architecture of FTHNet is shown in Fig. 4, which consists of 4 parts: the Transformer Backbone, the Distortion Perception Network, the Parameter Hypernetwork, and the Target Network.

The Transformer Backbone contains a patch embedding layer and four stages in different resolutions. Each stage comprises several Basic Transformer Blocks (BTBs) and a downsampling layer. **Firstly**, assuming an input fundus image, $\textbf{I}_{in} \in \mathbb {R}^{H\times W \times 3}$, the Transformer Backbone exploits a non-overlapping patch embedding layer consisting of a $4\times 4$ convolution (*conv*) to extract shallow feature $\textbf{I}_{0} \in \mathbb {R}^{\frac{H}{4}\times \frac{W}{4} \times C}$. **Secondly**, 4 stages are used for feature extraction on $\textbf{I}_{0}$. We adopt a $4 \times 4$ *conv* with stride 2 as the downsampling layer to the feature maps and double the channel dimension. Thus, the feature of the *i*-th stage is denoted as $\textbf{X}_{i} \in \mathbb {R}^{\frac{H}{4\times 2^i} \times \frac{W}{4\times 2^i} \times 2^{i}C}$. Here, *i* = 0, 1, 2, 3 indicates the four stages.

We design the Distortion Perception Network to extract distorted information, as shown in Fig. 4(b). Each stage input is processed in the Distortion Perception Block (DPB). The input feature maps are $\textbf{X}_{i}, i=0,1,2,3$. Then, we get four distorted information vectors through the DPB. Finally, distorted information vectors are concatenated to obtain the semantic vector $\textbf{V} \in \mathbb {R}^{L \times 1}$.

The parameters of the Target Network are generated from the Parameter Hypernetwork shown in Fig. 4(c). We design five stages for processing the parameters: one $1\times 1$ *conv* layer for channel merging and one Parameter Generating Layer (PGL). The Parameter Hypernetwork gets input directly from the final stage of the Transformer Backbone. The input feature map is denoted as $\textbf{X}_{3} \in \mathbb {R}^{\frac{H}{32} \times \frac{W}{32} \times 8C}$. We adopt a $1\times 1$ *conv* layer to merge channels by half. After channel merging, the feature of the *i*-th stage is denoted as $\textbf{P}_{i} \in \mathbb {R}^{\frac{H}{32} \times \frac{W}{32} \times \frac{8c}{2^i}}$. Here, *i* = 0, 1, 2, 3, 4 indicates the five stages. Then, the Parameter Hypernetwork exploits the PGL to generate the weights and biases of the linear layers for the Target Network. The weights and bias are denoted as $\textbf{WP}_{i}$and $\textbf{BP}_{i}$.

The Target Network consists of five linear layers. The input is the $\textbf{V}$ from the Distortion Perception Network. The weights and biases of the Target Network are from the Parameter Hypernetwork. We utilize five linear layers to generate the predicted fundus image quality scores.

In the implementation, we change the patch embedding channel *C* and the combination ($N_1, N_2, N_3, N_4$) in the Transformer Backbone to establish two different FTHNet models: FTHNet-S: (2,4,6,2), *C*:32; FTHNet-L: (2,4,6,2), *C*:64.

#### Basic transformer block

The emergence of Transformer provides an alternative to address the limitations of CNN-based methods in modeling non-local self-similarity and long-range dependencies. However, the computational cost of the standard global Transformer is quadratic to the spatial size of the input feature (HW). Therefore, to avoid this problem, we apply the transformer blocks with the Window-based Multi-head Self-Attention (W-MSA)^32^ in the Transformer Backbone. The computational complexity of W-MSA is linear to the spatial size, which is more wieldable than standard global MSA. We also add the alternate window-shifting operation in the BTB to introduce cross-window connections. The BTB consists of one W-MSA, one Multilayer Perceptron (MLP), and two normalization layers, as shown in Fig. 4(e). BTB can be formulated as eq.2.

2 $$\begin{aligned} \begin{aligned}&\textbf{F}^{\prime } = \text {W-MSA}(\text {LN}(\textbf{F}_{in}))+\textbf{F}_{in}, \\&\textbf{F}_{out} = \text {MLP}(\text {LN}(\textbf{F}^{\prime }))+\textbf{F}^{\prime }, \end{aligned} \end{aligned}$$

where $\textbf{F}_{in}$ represents the input feature maps of a BTB. $\text {LN}(\cdot )$ represents the layer normalization. $\textbf{F}^{\prime }$ and $\textbf{F}_{out}$ denote the output feature of W-MSA and MLP respectively.

where Concat$(\cdot )$ denotes the concatenating operation and $\textbf{W}^{O}\in \mathbb {R}^{{C \times C}}$ are learnable parameters. We reshape $\textbf{X}^{i}_{o}$ to obtain the output window feature map $\textbf{X}^{i}_{out} \in \mathbb {R}^{{L \times L \times C }}.$ Finally, we merge all the patch representations $\{\textbf{X}_{out}^{1}, \textbf{X}_{out}^{2},\textbf{X}_{out}^{3}, \cdots , \textbf{X}_{out}^{N} \}$ to obtain the output feature maps $\textbf{X}_{out} \in \mathbb {R}^{{H \times W \times C }}$.

**Multilayer Perception**

The Multilayer Perception(MLP) in the BTB resembles the most utilized methods in Transformers, consisting of a linear layer with a GELU activation, two dropout layers, and another linear layer.

#### Distortion perception block

We design the DPB in the Distortion Perception Network to extract the distortion information from the feature maps in different resolutions. Distortions of the fundus images are widely distributed on different scales. For example, spots and flares affect only some small areas; over-darkness and over-exposure influence the whole image. Rather than dealing with the final feature map, the DPBs get inputs from four backbone stages at different resolutions.

As depicted in Fig.. 4(f), the DPB consists of one $1\times 1$ *conv* layer, one SoftPool^33^ layer, and one linear layer. We adopt the $1\times 1$ *conv* and SoftPool to merge the channels and to downscale the spatial size of feature maps, respectively. The DPB can be formulated as follows:

3 $$\begin{aligned} \begin{aligned}&\textbf{X}^{\prime } = \text {SoftPool}(1\times 1 ~ \text {Conv}(\textbf{X})), \\&\textbf{V} = \text {Linear}(\text {Flatten}(\textbf{X}^{\prime })), \end{aligned} \end{aligned}$$

where $\textbf{X} \in \mathbb {R}^{H \times W \times C}$ are the input feature map of a DPB. $\text {SoftPool}$, $1\times 1 ~ \text {Conv}$, $\text {Linear}$, and $\text {Flatten}$ represents the SoftPool layer, the $1\times 1$ *conv* layer, the linear layer, and flatten operation, respectively. $\textbf{X}^{\prime } \in \mathbb {R}^{\frac{H}{12} \times \frac{W}{12} \times \frac{C}{8}}$ denotes the output feature. $\textbf{V} \in \mathbb {R}^{l \times 1}$ is the distortion information vector.

#### Parameter generating layer

The parameters of the Target Network are processed by the Parameter Generating Layer (PGL) shown in Fig. 4(g). The PGL consists of two branches, one for generating the weight parameters and another for generating the bias parameters. The weight branch is formulated as follows:

4 $$\begin{aligned} \textbf{WP} = \text {Reshape}(3\times 3 ~ \text {Conv}(\textbf{P})) \end{aligned}$$

Where $\textbf{P}$ denotes the input feature map of PGL. $\textbf{WP}$ represents the weight parameter. $3\times 3 ~ \text {Conv}$ and $\text {Reshape}$ represent the $3 \times 3$ *conv* layer and reshape operation respectively.

Similarly, the bias branch can be formulated as follows:

5 $$\begin{aligned} \textbf{BP} = \text {Linear}(\text {SoftPool}(\textbf{P})) \end{aligned}$$

where $\textbf{BP}$ represents the weight parameter. $\text {Linear}$ and $\text {SoftPool}$ denote the linear layer and SoftPool layer respectively.

Though this process seems complicated, it can directly generate the parameter matrix with desired shape and length if we calculate the output channel using $\frac{Channel_{in}\times H\times W}{\frac{L}{2^{i-1}} \times \frac{L}{2^i}}$, where $H\times W$ represents the size of the feature map, and *L* represents the input length of Target Network in the *ith* stage.

#### Loss function

We choose the smooth L1 loss^34^ as our model’s loss function. To be specific, the smooth L1 loss is formulated as

6 $$\begin{aligned} \mathscr {L}_{smoothL1} = {\left\{ \begin{array}{ll} |\textbf{y}-\textbf{y}^{\prime }|-0.5, & otherwise \\ 0.5\times (\textbf{y}-\textbf{y}^{\prime })^2, & -1<\textbf{y}-\textbf{y}^{\prime }<1 \\ \end{array}\right. } \end{aligned}$$

where $\textbf{y}$ denotes the ground-truth fundus image quality score, $\textbf{y}^{\prime }$ denotes the predicted score.

### Metrics

We chose root mean square error (RMSE), Pearson correlation coefficient (PLCC), and Spearman’s rank correlation coefficient (SRCC) as the metrics to evaluate the performance of our model.

The RMSE in eq. 7 shows the deviation between the prediction and target values. The lower the RMSE, the more robust the model in prediction.

7 $$\begin{aligned} RMSE = \sqrt{\frac{1}{n}\sum _{i=1}^{n}(y^{\prime }_{i}-y_{i})^2} \end{aligned}$$

where $y_{i}$ denotes the ground-truth MOS, $y^{\prime }_{i}$ denotes the predicted MOS.

The PLCC and SRCC are both standard metrics applied in the IQA works. The PLCC shown in eq.8 varies from −1 to 1 and shows the correlation between prediction and target values. The higher PLCC means the model’s predictions are closer to the images’ actual scores.

8 $$\begin{aligned} PLCC = \frac{\sum _{i}(y^{\prime }_i-\bar{y}^{\prime })(y_i-\bar{y})}{\sqrt{\sum _i(y^{\prime }_i-\bar{y}^{\prime })^2\sum _i(y_i-\bar{y})^2}} \end{aligned}$$

where $\bar{y}^{\prime }$ represents the average value of $y^{\prime }_{i}$ and $\bar{y}$ represents the average value of $y_{i}$.

The SRCC in eq.9 varies from −1 to 1 and measures the monotonicity of the models’ prediction.

9 $$\begin{aligned} SRCC = 1 - \frac{6\sum ^{N}_{i=1}{d_{i}^2}}{N(N^2-1)} \end{aligned}$$

where $d_{i} = y^{\prime }_{i} - y_{i}$ denotes the difference between $y^{\prime }_{i}$ and $y_{i}$.

### Availability of data and materials

The codes of training and validating are available at https://github.com/HudenJear/BasiQA. The code for dataset collecting and labeling is available at https://github.com/HudenJear/FIQA-GUI-Labeling. The data that is used in this research can be accessed at https://figshare.com/articles/dataset/FIQS_Dataset_Fundus_Image_Quality_Scores_/28129847?file=51531041. Please contact the corresponding author if there are any questions about data and code.

## Results

### Implementation details

The train, test, and validation subsets are split proportionately 80%, 15%, and 5% randomly in each round, and 10-round cross-validation is applied in training. During the training procedure, Fundus images are resized to $384 \times 384$. The Adam^35^ optimizer is adopted. We also apply data augmentation consisting of horizontal/vertical flip and random crop. The learning rate is set to $0.5 \times 10^{-4}$ with linear annealing, and the batch size is set to 16. We use PyTorch 1.9 and CUDA 11.2. Every model is trained for 120000 iterations with a warming-up of 1000 iterations, equivalently 853 epochs. It takes about 12 hours to use an NVIDIA RTX3090 GPU to finish the training process.

**Table 1:** Quantitative comparisons with SOTA algorithms on our FQS dataset.

Method	SRCC	PLCC	RMSE	Params(M)
TRIQ^15^	0.4153	0.4327	–	23.68
DeepIQA^12^	0.2964	0.2798	–	6.29
HyperIQA^13^	0.9351	0.9305	–	28.28
GraphIQA^14^	0.9280	0.9355	–	51.52
SaTQA^36^	0.9145	0.9256	–	50.55
BRISQUE^37^	0.9020	0.9220	8.039	–
ILINIQE^38^	0.6550	0.7191	35.46	–
BIQI^39^	0.5788	0.5492	76.2	–
DIVINE^40^	0.1566	0.1686	43.19	–
FTHNet-S (Ours)	0.9330	0.9435	7.118	**5.662**
**FTHNet-L (Ours)**	**0.9358**	**0.9442**	**7.024**	14.88

### Comparisons with state-of-the-art methods

To evaluate the performance of the proposed FTHNet, we compare our FTHNet with several SOTA methods. These methods include four model-based methods (BRISQUE^37^, ILINIQE^38^, BIQI^39^, and DIVINE^40^). Meanwhile, 5 deep learning-based methods: DeepIQA^12^, HyperIQA^13^, GraphIQA^14^, SaTQA^36^, and TRIQ^15^, are also included in the comparison methods. These methods encompass a comprehensive range from algorithm-based methods to the newest deep learning method leveraging the Transformer and contrastive learning.

As shown in the Tabel.1, The model-based methods mentioned above can merely reach the performance of FTHNet, though these methods are retrained on our FQS dataset. Since the model-based methods do not have network parameters and computing flops, the corresponding data is not shown in the table.

The IQA methods had not been applied to FIQA tasks before this work, and we retrained HyperIQA, GraphIQA, and TRIQ for their best performance in the comparison study. The quantitative comparisons on our FQS dataset are shown in Table.1. Our best model, FTHNet-L, achieves 0.0133, 0.0183, 0.5161, and 0.6690 improvements in PLCC compared to GraphIQA, HyperIQA, TRIQ, and DeepIQA. Meanwhile, FTHNet-L has 0.0143, 0.0072, 0.5270, and 0.6459 better performance in SRCC, respectively. These improvements of FTHNet-L are significant enough for IQA tasks. Furthermore, although FTHNet-L is already smaller than most other models, our lightweight model, FTHNet-S, outperforms other methods by 0.008, 0.013, 0.5108, and 0.6637 in PLCC, respectively, while requiring 5.67 M Params.

**Table 2:** Ablation study of Transformer Backbone, window shifted operation(WSO), loss function, downsampling structure, and hypernetwork structure.

Type	Method	SRCC	PLCC	RMSE	Params(M)	FLOPS
Backbones	ResNet^41^	0.1672	0.1630	39.24	27.97	12.70 G
	ConvNeXt-L^42^	0.8095	0.8081	12.19	55.25	26.30 G
	MSG Transformer^43^	0.9241	0.9277	7.615	33.29	16.02 G
	**BTB**	**0.9358**	**0.9442**	**7.024**	**14.88**	**6.044 G**
WSO	w/o	0.9263	0.9405	7.356	14.88	6.044 G
	with	**0.9358**	**0.9442**	**7.024**	14.88	6.044 G
Loss	$\mathscr {L}_1$	0.93245	0.9439	6.716	14.88	6.044 G
	$\mathscr {L}_2$	**0.9363**	0.9389	7.028	14.88	6.044 G
	$\mathscr {L}_1 + \mathscr {L}_2$	0.93405	0.9408	6.914	14.88	6.044 G
	$\mathscr {L}_{smoothL1}$	0.9345	**0.9447**	**6.581**	14.88	6.044 G
Downsampling Structure	Direct	**0.9357**	0.9426	**6.978**	0.572	82.39 M
	Stepwise	0.9354	**0.9437**	6.988	**0.393**	**56.67 M**
Hypernetwork	w/o	0.6092	0.6077	20.93	**12.28**	**5.06 G**
	with	**0.9358**	**0.9442**	**7.024**	14.88	6.044 G

**Fig. 5:**
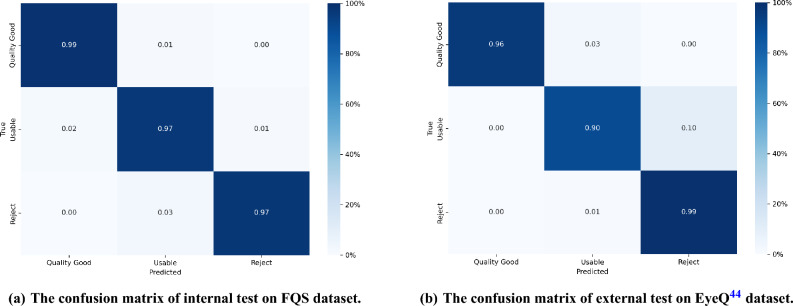
The confusion matrices of internal and external dataset test. We applied our pretrained model to (**a**) the FQS dataset and (**b**) EyeQ^44^ dataset. The image quality level is decided by its quality score prediction following the same criteria as in Section.1.1.2. The FTHNet achieves great performance in quality level classification on internal dataset validation. The external test also indicates that FTHNet works effectively on the external dataset. The results of classification show great precision and generalizability of our FTHNet.

We also evaluated the performance of our model on a 3-level quality classification task using the FQS dataset. Although the FTHNet model is designed to provide a continuous quality score, this classification task provides a simplified metric for internal validation. The images were categorized into three quality levels based on their predicted scores, using the same criteria described in Section 1.1.2. The results, presented in Fig. 5a, show that very few images fell in incorrect classification. This analysis confirms that the FTHNet model exhibits strong performance on internal validation.

### External validation

To perform the external validation, we conducted a complementary experiment using the heterogeneous EyeQ dataset^44^. This evaluation was designed to assess the generalizability of our model on an independent dataset. The 28,790 images from the EyeQ dataset were processed by our model to obtain quality score predictions. These predictions were then converted to quality levels using the same criteria outlined in Section 1.1.2 and compared against the ground-truth labels to calculate the performance metrics.

The results, presented in Fig. 5b, demonstrate that FTHNet achieves good performance on external data validation, confirming its generalizability. We observed a slightly higher error rate in the “Usable” category, which may be attributed to differences in the quality assessment criteria between our dataset and the EyeQ dataset.

### Ablation study

#### Transformer backbone

To analyze the effect of the Transformer Backbone, we compare our BTB with three solid Transformer Backbones in Table. 2, including two CNN-based backbones (ResNet^45^ and ConvNeXt^42^) and one Transformer-based backbone(MSG-Transformer^43^) with other structure of FTHNet unchanged. It can be observed in the table that our proposed BTB achieves the best performance.

#### Window shift operations

The window shift operations are designed to augment the information exchange between adjacent windows in the BTBs. We conducted the ablation study to analyze the effect of the window shift operations. The results are reported in Table. 2. The results indicate that the window shift operations can build cross-window connections and improve the performance of FTHNet.

#### Depth and patch embedding channel

We explore the effect of different patch embedding channels *C* and combinations ($N_1, N_2, N_3, N_4$) in the extraction backbone on model performance after choosing the BTB with W-MSA as the extraction block. The results are shown in Table. 3.

**Table 3:** Ablation study of combination ($N_1, N_2, N_3, N_4$) and patch embedding channel(*C*).

($N_1,N_2,N_3,N_4$)	*C*	SRCC	PLCC	RMSE	Params(M)
(2, 2, 2, 2)	32	0.9328	0.9409	7.328	**4.748**
(2, 2, 2, 2)	64	0.9355	0.9418	7.127	11.70
(2, 2, 6, 2)	32	0.9331	0.9439	7.094	5.558
**(2, 2, 6, 2)**	**64**	**0.9358**	**0.9442**	7.024	14.88
(2, 2, 6, 2)	96	0.9332	0.9404	7.156	30.34
(2, 4, 6, 2)	32	0.9303	0.9424	7.161	5.662
(2, 4, 6, 2)	64	0.9338	0.9437	**6.962**	15.28
(2, 4, 6, 2)	96	0.9312	0.9430	6.971	31.23
(2, 2, 12, 2)	64	0.9323	0.9415	7.112	19.64
(2, 2, 12, 2)	96	0.9279	0.9395	7.039	41.02
(2, 2, 18, 2)	64	0.9356	0.9416	7.266	24.40
(2, 2, 18, 2)	96	0.9314	0.9374	7.2975	51.71

It can be observed that our FTHNet-L ((2, 2, 6, 2), *C*:64) can achieve the best performance. Compared with other patch embedding channels, 64 is the optimal choice. Considering the performance and model parameters, we finally chose ((2, 4, 6, 2), *C*:32) as our FTHNet-S.

#### Downsampling structure

We conduct this ablation study to explore the effect of downsampling structure in the Parameter Hypernetwork. Two downsampling structures are available in the Parameter Hypernetwork: stepwise and direct downsampling. As shown in Table. 2, compared with the direct structure, the stepwise downsampling structure achieves almost the same performance with fewer model parameters and computational complexity. Thus, we choose the stepwise downsampling structure in our FTHNet.

#### Loss function

We test different loss functions, including L1 loss, L2 loss, and Smooth L1 loss^34^, while training the models for better performance. The L1 loss is the most common function in the IQA. In addition, L2 loss and Smooth L1 loss are also applied in the IQA.

As listed in Table.2, compared with other loss functions, Smooth L1 loss achieves the best performance in PLCC and RMSE. Meanwhile, Smooth L1 loss gets the second-best performance in SRCC. Thus, we choose the Smooth L1 loss as the loss function of our FTHNet.

## Discussion

### Deployment experiment

**Fig. 6:**
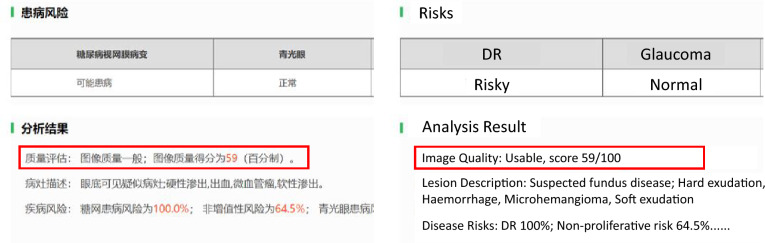
Implementation of the FTHNet in the diagnosis system. The FTHNet works in the emphasized region.

We deploy our FTHNet as an Application Program Interface (API) on a GPU server and bind our FTHNet with a new automatic diagnosis system. The implementation of the FTHNet in the diagnosis system is demonstrated in Fig. 6. This system can process fundus images and provide ophthalmologists with pathology information and the reliability of images. If the score of one image is high, the pathology information distinguished by the system shall be more trustworthy. The ophthalmologists utilizing this system can choose how to use the result based on the score of the FTHNet.

### Potential of real-time assessment

The inference speed is one of the most critical factors affecting the application of our FTHNet in clinical diagnosis. We deploy our FTHNet as an API on a GPU server to test the inference time of our FTHNet, and the results are in Table.4. The single test means the average inference time of a single fundus image.

**Table 4:** Evaluation time consumption of different FTHNet models.

Methods	Params(M)	FLOPS(G)	Single Test(ms)
**FTHNet-S**	**5.662**	**1.981**	**44.45**
FTHNet-L	14.88	6.044	56.31

We can observe from Table.4 that the inference time of FTHNet-S is 44.45 ms, and even FTHNet-L is only 56.31 ms. With the short inference time, our FTHNet can provide real-time assessment in clinical diagnosis.

### Effect of hypernetwork structure

In this subsection, we discuss the effect of the hypernetwork structure on FIQA tasks.

To explore the importance of hypernetwork structure in the FIQA task, we conduct this experiment and show the results in Table.2. Note that *w*/*o* *hyper* *network* means the parameters of the Target Network are learned by backpropagation rather than provided by the hypernetwork.

It can be observed that the performance of FTHNet improves significantly with the hypernetwork structure despite a slight increase in model parameters. It indicates the importance of hypernetwork structure in the FIQA tasks. Our FTHNet is constructed based on the hypernetwork structure. The Target Network has flexible parameters from the hypernetwork varying with the input image, which is essential for the quality assessment with fundus images of complex and diverse degradation.

**Fig. 7:**
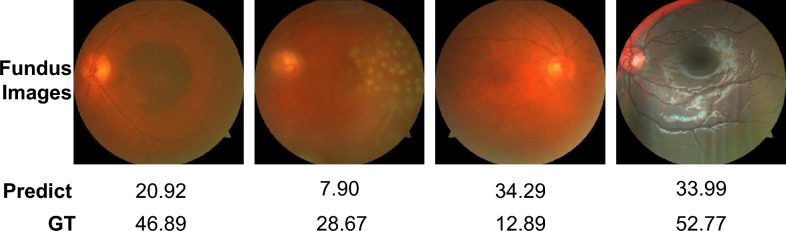
Failed cases of FTHNet on our FQS dataset. Most of these failed cases are from the “Reject” category and have multiple features of different degradation. More images of combined degradation will be included in the FQS dataset to increase the generalizability and robustness of the FTHNet.

### Failure cases

Although FTHNet demonstrates robust overall performance, an analysis of its failure cases reveals limitations under specific, challenging conditions. Fig. 7 illustrates instances from our FQS dataset where FTHNet’s prediction values exhibit a deviation when compared to the ground truth. Further observation indicates that these failure cases predominantly belong to images in the “Reject” category.

These shown failure cases are frequently associated with complex degradation profiles, primarily combinations of severe blur and dense haze. Such coexistence of severe degradations is relatively rare within the current FQS dataset. The sample number for these combined degradation categories may influence model generalizability. Still, for most of these instances, while the predicted MOS value may be inaccurate, their quality scores are within the range of the “Reject” quality category. The limited exposure to such specific degradation combinations degrades the precision of the quality score prediction for these failure cases. A systematic augmentation of our training dataset by incorporating more examples of these complex and rare degradation types may improve performance. Increasing the representation of these challenging cases is expected to enhance the model’s robustness and generalizability.

Therefore, a key direction for our future work involves the enrichment and improvement of the FQS dataset. We plan to specifically augment the dataset with more examples containing images of combinations of severe degradations. This will be crucial for enhancing FTHNet’s robustness and precision of prediction for images at the lowest end of the quality scale. We acknowledge the potential benefits of addressing the data imbalance, specifically the underrepresentation of severely degraded images. As a future direction, we plan to investigate the use of oversampling techniques during model training. We believe that improving FTHNet’s robustness to unbalanced data distributions will also be effective in minimizing failure cases and further enhancing its performance.

## Conclusions

For traditional FIQA methods, most give classification prediction; it is difficult to compare results within each category; algorithms are old and not deployed in practice. However, our FTHNet is trained to give continuous quality scores, which are intuitive and convenient to compare; it is lightweight and easy to deploy in automatic diagnosis systems; it has a new transformer-based hypernetwork architecture and leading performance among IQA methods.

Our extensive experimental validation has demonstrated FTHNet’s leading performance in FIQA, consistently outperforming a range of existing methods. Furthermore, ablation studies have systematically validated the efficacy of FTHNet’s individual architectural components. However, an analysis of failure cases has also highlighted areas for continued advancement. Specifically, FTHNet’s performance is less robust for certain rare and complex combinations of severe degradations, primarily due to their underrepresentation in the current iteration of our FQS dataset. Our future work will therefore concentrate on two key areas. First, we will expand the proposed FQS dataset by collecting more data, specifically targeting these complex cases. Second, we will focus on enhancing FTHNet’s robustness to unbalanced data distributions. This will involve investigating more advanced training techniques, such as oversampling the underrepresented classes, to further minimize failure cases and improve overall model performance.

To facilitate continued research and reproducibility, we have publicly released the source code for FTHNet and the complete FQS dataset with detailed annotations (as specified in the Availability of Data and Materials section). Additionally, tools developed for constructing the FQS dataset are also made available to support the broader fundus image research community. We hope this work can serve as a baseline for FIQA and benefit the fundus image research by providing a new quantitative metric in the future.
